# Translational Research and Implementation of Solutions for Aging: A Call to Action

**DOI:** 10.3389/ijph.2025.1607768

**Published:** 2025-04-29

**Authors:** Alan Hipólito Juárez Solano

**Affiliations:** ^1^ Graduate Program in Pharmacobiological Sciences, Faculty of Chemical Sciences, Autonomous University of San Luis Potosí, San Luis Potosí, Mexico; ^2^ Translational Science, Laboratory 4, Aging Research Center at the Center for Research and Advanced Studies, Mexico City, Mexico

**Keywords:** traslational research, traslational science, aging, basic science, scientific collaboration

**The IJPH series “Young Researcher Editorial” is a training project of the Swiss School of Public Health**

As populations age, governments of high-income countries are focused on innovation and providing expensive therapies to their citizens, while governments of low- and middle-income countries struggle to overcome limitations in basic infrastructure [1]. This disparity is particularly evident in aging-related health issues, where the complexity of these conditions adds further challenges to translating scientific discoveries into clinical applications.

The gap between basic research and its clinical application persists due to various factors. The lack of connection between researchers and healthcare professionals creates communication barriers that hinder the practical implementation of promising discoveries [2]. Regulatory frameworks, particularly in emerging fields like stem cell therapy, often require extensive safety documentation and multiple phases of clinical trials, delaying the arrival of innovations to market by 5–10 years [3]. Furthermore, the scarcity of funding for translational research varies significantly by region: while the EU and the U.S. have dedicated programs for this research, many developing nations lack specific support for the implementation of new technologies in clinical practice [4]. Additionally, the absence of translational programs focused on aging in the training of healthcare professionals further complicates the integration of scientific advances into daily medical practice.

Recent advancements in translational research underscore the urgent need to bridge the gap between basic science and clinical applications, particularly in diseases associated with aging. While therapies like neural stem cell transplantation for Parkinson’s disease have reached Phase I clinical trials and shown promise in improving cognitive and motor function, their full potential can only be realized if fundamental research findings are efficiently translated into medical practice [5] However, significant challenges persist, including ensuring cell survival, managing immune responses, and navigating complex regulatory approval processes, all of which delay the integration of scientific advancements into patient care.

Another relevant case is the connection between gut microbiota and Alzheimer’s disease, where findings from basic research must be effectively translated into clinical practice worldwide. This relationship has advanced from preclinical and observational studies to clinical trials exploring the therapeutic potential of specific bacterial strains and metabolites in slowing cognitive decline [6]. Initial results suggest promising applications. but large-scale clinical validation remains a challenge. Factors such as microbiome variability and individual patient characteristics complicate the process, highlighting the need for strategies that facilitate the integration of these discoveries into routine medical care, regardless of geographic or economic constraints.

The previous examples allow for an understanding of the advances and complications represented by both therapeutic strategies. Furthermore, they also highlight the difficulties associated with the implementation of these technologies in clinical practice.

To bridge the gap between research and its clinical application, it is essential to implement targeted strategies that do not depend on highly specialized infrastructure and can be adapted to different contexts. A key measure is the incorporation of translational research modules in medicine and nursing curricula, with a focus on geriatric care and the clinical application of new therapies [7]. Additionally, structured interdisciplinary collaborations should be established, based on successful models from other medical areas. For example, in oncology, the implementation of tumor boards—where pathologists, oncologists, surgeons, and researchers work together to evaluate clinical cases—has significantly improved treatment personalization. Similarly, in cardiovascular medicine, heart team conferences allow for joint decision-making between cardiologists, cardiovascular surgeons, and scientists, optimizing patient outcomes.

For neurodegenerative diseases, specialized centers in translational research should be developed that integrate basic scientists, neurologists, and pharmaceutical industry experts, accelerating the transition from laboratory discoveries to viable clinical treatments. Most importantly, these models do not require costly technologies or advanced infrastructure to be effective. Strategies such as forming collaborative networks, conducting interdisciplinary meetings, and training in translational research can be implemented in diverse health systems, even in countries with limited resources. Applying these approaches to aging care would facilitate the integration of new medical interventions, ensuring that scientific advances benefit a larger population globally [8].

Addressing the challenges of aging populations requires urgent, coordinated action from researchers, healthcare professionals, and policymakers. Establishing translational research committees as a core component of healthcare strategies, backed by dedicated funding and strong implementation frameworks, is essential. Additionally, integrating translational science programs across academic levels will foster collaboration and accelerate the application of scientific advances in clinical practice, ultimately reducing aging-related diseases and enhancing the quality of life for older adults.

## Data Availability

The original contributions presented in the study are included in the article/supplementary material, further inquiries can be directed to the corresponding author.
